# Carrier of Wingless (Cow), a Secreted Heparan Sulfate Proteoglycan, Promotes Extracellular Transport of Wingless

**DOI:** 10.1371/journal.pone.0111573

**Published:** 2014-10-31

**Authors:** Yung-Heng Chang, Yi Henry Sun

**Affiliations:** 1 Graduate Institute of Life Sciences, National Defense Medical Center, Taipei, Taiwan, Republic of China; 2 Institute of Molecular Biology, Academia Sinica, Taipei, Taiwan, Republic of China; University of Dayton, United States of America

## Abstract

Morphogens are signaling molecules that regulate growth and patterning during development by forming a gradient and activating different target genes at different concentrations. The extracellular distribution of morphogens is tightly regulated, with the *Drosophila* morphogen Wingless (Wg) relying on Dally-like (Dlp) and transcytosis for its distribution. However, in the absence of Dlp or endocytic activity, Wg can still move across cells along the apical (Ap) surface. We identified a novel secreted heparan sulfate proteoglycan (HSPG) that binds to Wg and promotes its extracellular distribution by increasing Wg mobility, which was thus named Carrier of Wg (Cow). Cow promotes the Ap transport of Wg, independent of Dlp and endocytosis, and this function addresses a previous gap in the understanding of Wg movement. This is the first example of a diffusible HSPG acting as a carrier to promote the extracellular movement of a morphogen.

## Introduction

Morphogens are signaling molecules that can be distributed in a developing tissue along a concentration gradient and affect development in a concentration-dependent manner. The formation and interpretation of the gradient are regulated at multiple levels.

The *Drosophila* morphogen Wingless (Wg) is one of the founding members of the Wnt family of signaling molecules. In *Drosophila* embryo and imaginal disc development, Wg has been shown to act as a long-range morphogen [1], [2], [3], [4]. In the best-studied wing disc, Wg is expressed in several rows of cells at the dorsoventral (D-V) boundary in the prospective wing pouch region [3]. Wg can be secreted from producing cells or localized extracellularly to form a concentration gradient to regulate target genes at different levels [5], [6]. Although Wg is secreted from the apical (Ap) surface of its producing cells [7], [8], extracellular Wg (exWg) is localized primarily on the basolateral (Ba) surface [5]. ExWg can be detected within a few rows of cells away from its producing cells at the Ap surface but spreads more than 20 cells away at the lateral surface [7]. These results suggest that the long-range movement of exWg occurs on the Ba surface. However, the mechanisms by which exWg moves short distances along the Ap surface and longer distances along the Ba surface remain unclear.

In receiving cells, Wg can also be found in puncta representing internalized Wg. The internalization of Wg is dependent on endocytosis and occurs at both Ap and Ba surfaces [5], [9], [10]. Whereas the secretion and degradation of Wg are dependent on dynamin, the movement or distribution of exWg is independent of endocytosis [5], [7], [9], [10], [11].

Wg distribution is affected by heparan sulfate proteoglycans (HSPGs), which are proteins modified by heparan sulfate (HS) glycosaminoglycan (GAG) chain attachments. Enzymes for GAG and HS synthesis, such as Sulfateless (Sfl) and Brother of tout-velu (Botv), are required for exWg distribution [12], [13], [14]. These results suggest that the exWg movement requires HSPGs. Within large *sfl* and *botv* mutant clones, although exWg is reduced, there is Wg accumulation within and behind the clone, suggesting that some HSPG from neighboring wild-type (WT) cells can act non-autonomously [12], [14], [15]. Because the two HSPGs known to affect Wg signaling, Dally and Dally-like (Dlp) (see below), are membrane-anchored, an unidentified diffusible HSPG is predicted to serve this role.

Dally and Dlp belong to the glypican family of HSPGs, and both can bind to Wg and are involved in regulating Wg distribution and signaling [14], [16]. The movement of Wg from the Ap to Ba surface was reported to be dependent on Dlp through transcytosis [7], although this finding was contradicted by another study [17]. Moreover, this vertical intracellular translocation of Wg does not explain the lateral intercellular spread of exWg. It has been proposed that Wg bound to Dlp can be transferred to adjacent Wg receptors, depending on the ratio of Dlp to DFz2 [16], [17], [18], although whether this mechanism transfers Wg to adjacent cells has not yet been demonstrated.

Extracellular movement of Wg at or near producing cells likely occurs independent of membrane-anchored Dlp and DFz2, as these levels are low in the Wg-producing cells at the D-V border [14], [15], [16], [19], [20]. Furthermore, *dlp*-null clones do not affect the exWg level in this region [14], which leaves open the question of what factor is responsible for moving Wg from its source to adjacent Dlp-expressing cells. Dally is present at high levels at the D-V border, but it plays only a minor role that is partially redundant with Dlp [5], [14], [16]. Even in a *dally dlp* double mutant clone, exWg is detected away from the producing cells [14]. The extracellular hydrolase Notum/Wingfull can modify Dlp to reduce its ability to bind and stabilize Wg, thereby reducing, rather than promoting, the range of Wg distribution [21], [22]. Therefore, some unidentified factor must be responsible for moving Wg away from its source.

In this study, we identified Carrier of Wg (Cow) as a novel secreted HSPG. Our results showed that Cow can bind to exWg to increase its rate of movement and stability. The identification of Cow answers four previously unknown aspects of Wg gradient formation. First, Cow is localized primarily at the Ap surface and is responsible for the Ap movement of Wg. Second, Cow is a diffusible HSPG, which can explain the non-autonomous rescue of Wg movement in clones defective for HS synthesis. Third, Cow is present at the D-V border and is responsible for moving Wg away from its source to interact with Dlp and receptors. Fourth, diffusible Cow can mediate the transfer of Wg to adjacent cells, a role not satisfactorily explained by membrane-anchored Dlp.

## Materials and Methods

### Transgenes

Human *Testican-2* cDNA was obtained from the Human Unidentified Gene-Encoded (HUGE) Large Proteins Database (Kazusa DNA Research Institute) and cloned into the *pUAST-flag* vector with a Flag tag at the N-terminal [23] to generate *UAS-Testican-2*. The *cow* cDNA was amplified by RT-PCR and cloned into *pUAST-flag* to generate *UAS-Cow*. The protein product does not contain the Flag tag because it is cleaved at the signal peptide. For Flag-tagged Cow, the Flag tag “DYKDDDDK” was inserted after the signal peptide of Cow to generate *UAS-SP-Flag-Cow*. For *SP-EGFP-Cow*, the EGFP replaced the Flag tag in *SP-Flag-Cow*. For *Cow-GPI*, the Dally GPI domain (aa577–626) was added to the C-terminus of Cow. The two putative GAG sites SG1 (ISGY) and SG2 (NSGN) were mutated to IAAY and NAAN, respectively, to produce *SP-Flag-Cow-mSG1+2*. The *Cow-miRNA* constructs were designed based on the method of Chen et al. [24]. The targeting sites of *Cow-miRNA-1* and *Cow-miRNA-2* were TTAGCATAGTTGCTGCGTAAGA in the 5′-untranslated region (UTR) and CCACAAGAATCGTGATGAGATA in the N region, respectively (see Figure S1B). *SP-Flag-Cow*, *SP-EGFP-Cow*, *SP-Flag-Cow-mSG1+2*, *Cow-miRNA-1* and *Cow-miRNA-2* were cloned into the *pUAST* vector [25]. The *Cow-dsRNA* construct was made by cloning the sense and anti-sense sequences of the Cow TY-C region into the *pWIZ* vector [26] at *Bgl*II/*Xho*I and *Nhe*I/*Xba*I sites, respectively. The detailed construction process is available upon request.

### RT-PCR

The primer sets for RT-PCR and the conditions for semi-quantitative analysis are available upon request.

### Fly stocks

*UAS-GFP-wg* [27], *UAS-DFz2* and *UAS-DFz2N* [28], *UAS-Dlp-HA* [16], *wg{KO; Gal4}* [29], *wg{KO; wg-HA}* [30], *wg^cx4^*, *sfl^03844^*, *Df(3R)Exel6193*, *Df(3R)BSC527*, *Df(3R)BSC619*, *Mi{ET1}CG13830^MB00767^*, *neur-lacZ* (*neur^A101^*), *UAS-Shi^ts^*, *UAS-lacZ*, *UAS-myrRFP*, *UAS-FLP*, *tub-p-Gal4*, *ap-Gal4*, *en-Gal4*, *hh-Gal4*, *ptc-Gal4*, *MS1096-Gal4*, and *nub-Gal4* were obtained from the Bloomington Drosophila Stock Center. The *cow^5Δ^* allele was generated by imprecise excision of the *Minos* transposable element *Mi{ET1}CG13830^MB00767^* from the 3′-UTR of *cow* (see Figure S1A).

### Immunostaining and *in situ* hybridization of the embryo and wing discs

Immunostaining and *in-situ* hybridization of the embryo and wing discs were performed following standard protocols [31], [32]. The primary antibodies included guinea pig anti-Sens (1∶1,000) [33], rat anti-Dll (1∶250) [3], goat anti-Dll (1∶100; Santa Cruz Biotechnology), rabbit anti-GFP (1∶250; Invitrogen), mouse anti-HA (1∶200; Abcam), mouse anti-β-Galactosidase (1∶500; Promega), rabbit anti-β-Galactosidase (1∶1,000; Cappel), mouse anti-Flag (1∶500; Sigma), mouse anti-Cut (1∶150), mouse anti-Wg (1∶200), and mouse anti-Ptc (1∶250), which were obtained from the Developmental Studies Hybridoma Bank. A polyclonal rabbit anti-Cow antibody was raised against the synthesized peptide Ac-YLEEEAKRRVNQQDNQDSQDC-amide (aa112–131) by Quality Controlled Biochemicals (Hopkinton, MA, USA). The extracellular staining protocol was performed as previously described [5], using 3× standard antibody concentrations. Secondary antibodies conjugated with FITC, Cy3 and Cy5 were purchased from Jackson ImmunoResearch. Some samples were co-stained with DAPI (1∶1,000; Sigma), FITC-Phalloidin (1∶200; Invitrogen), Texas Red-X-Phalloidin (1∶200; Invitrogen) and Alexa Fluor 680-Phalloidin (1∶200; Invitrogen). For comparative purposes, wing discs were dissected, fixed and stained in parallel and imaged under identical conditions. Images were collected with Zeiss LSM510 META and LSM780 confocal microscopes.

### Embryo cuticle and adult wing preparation

Embryo cuticle preparation was performed according to a published protocol [16], and samples were mounted in Hoyer's solution and photographed under dark-field microscopy. To examine the chemosensory bristle number, adult wings were mounted in Hoyer's solution and photographed under differential interference contrast (DIC) and phase-contrast microscopy.

### Generation of germline and mutant clones

Generation of the germline clone was performed according to the described protocol [34]. *hs-FLP/Y; FRT82B ovo^D1^/TM3, Sb* males were crossed to *hs-FLP; FRT82B cow^5Δ^/TM3, Sb* virgins. Progeny at the third instar were heat shocked at 37°C for 1 h once on day 5 and once on day 6. The *hs-FLP; FRT82B cow^5Δ^/FRT82B ovo^D1^* females were crossed to F*RT82B cow^5Δ^/TM3, Ser, twi-GFP* males to generate embryos that were maternal and zygotic *cow^5Δ^* mutants. To generate the *cow^5Δ^* mutant clone in wing disc and adult wing, the *ap-Gal4/UAS-FLP; FRT82B cow^5Δ^/FRT82B ubi-GFP Minute* was used.

### Immunoprecipitation and western blotting

The Cow protein levels in WT and *cow^5Δ^* homozygous embryos were analyzed by western blot. *cow^5Δ^* was balanced with the *twist-GFP*, and the non-GFP homozygous *cow^5Δ^* embryos were selected by detecting the level of Cow protein. WT and *sfl^03844^* homozygous embryos were selected and analyzed for HS modification of Cow protein. *sfl^03844^* was also balanced with *twist-GFP*, and non-GFP *sfl^03844^* homozygous embryos were collected and analyzed for the HS modification state of Cow. Coimmunoprecipitation of Wg and Cow was performed following a published protocol [20]. To address physiological interactions between GFP-Wg and SP-Flag-Cow, GFP-Wg and SP-Flag-Cow were coexpressed in embryos using *tub-Gal4* and then collected for immunoprecipitation assays. To examine endogenous Wg-HA and Cow protein interactions, protein extracts containing endogenous Wg-HA and Cow proteins were collected from WT, *wg{KO; Gal4}* and *wg{KO; wg-HA}* at the larval stage and then analyzed using the immunoprecipitation protocol. The primary antibodies used included mouse anti-HA (Roche), mouse anti-Flag (Sigma) and mouse anti-α-tubulin (Sigma). Light chain-specific secondary antibodies included goat anti-mouse and goat anti-rabbit conjugated with horseradish peroxidase (HRP) (Jackson ImmunoResearch).

### Extracellular binding assays for Cow with Wg and Testican-2 with Wnt5a

To test whether secreted Cow and Wg could interact in the extracellular space, the empty UAS vector *UAS-HA-Wg* [22] and *UAS-SP-Flag-Cow* were separately co-transfected with the *actin-Gal4* plasmid in S2 cells. After 48 h, the culture supernatants were separately collected. Five hundred micrograms of protein from each supernatant was mixed in different combinations and analyzed by immunoprecipitation as described above. In the binding assay using secreted Testican-2 with Wnt5a, the empty *pAC* vector, *pAC-SP-Flag-Testican-2* and *pAC-Wnt5a-HA* [35] (with the *Wnt5a* in the *pcDNA* moved to the *pAC* vector) were separately transfected into S2 cells and tested as described above.

### Wg protection assay

S2 cells were transfected with the HA-Wg vector for 48 h. The HA-Wg conditioned medium was added to replace the medium of empty or *Cow-dsRNA* vector-transfected cells. The HA-Wg content from the culture supernatant at different time points was measured by western blotting.

### Intensity plot for exWg

Staining for exWg in wing discs was performed according to the protocol of Strigini and Cohen (2000) [5]. Each plotting line was derived from a region of 600×650 pixels in the center of the wing pouch region to minimize the effect of the curvature of the D-V border. There are 600 single-pixel lines perpendicular to the D-V border; each 10 adjacent lines were averaged, and the 60 averaged lines were averaged to produce the intensity plot from one disc. The results from three discs were then averaged to obtain the intensity plot for each condition.

### Fluorescence recovery after photobleaching (FRAP)

The *dpp>GFP-Wg+lacZ* and *dpp>GFP-Wg+Cow* third instar wing discs were analyzed following the published procedure [6]. The time-lapse images were collected using a Zeiss LSM710 confocal microscope.

## Results

### Cow is a secreted HSPG

**Figure 1:**
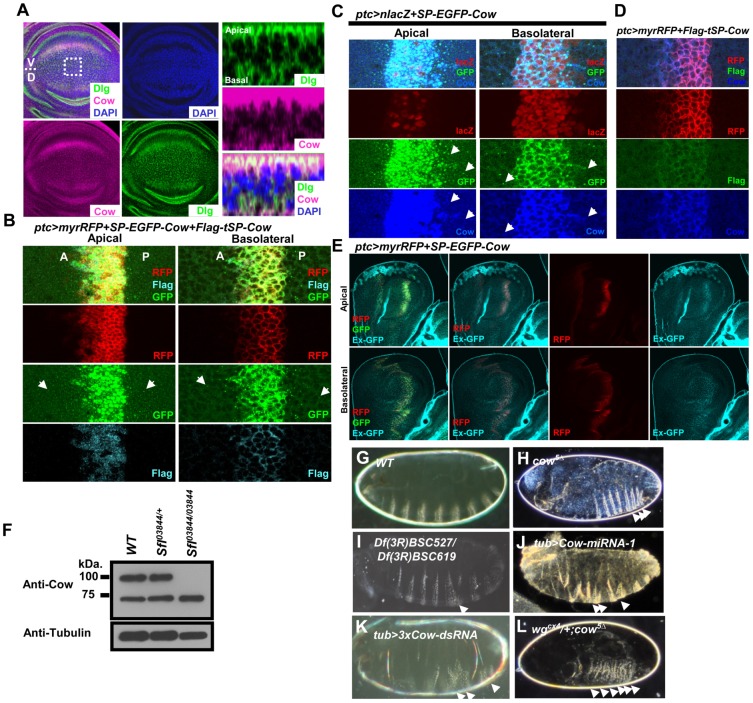
Cow is a secreted HSPG and genetically interacts with *wg*. (A) Cow protein (stained with anti-Cow, magenta) is detected throughout the wing disc. Dlg, green; DAPI, blue. Z-sections show that Cow is located primarily at the Ap surface but is also present at the Ba surface. In this and all subsequent figures on wing discs, the V side is located at the top, and the P side is at the right. (B) *SP-EGFP-Cow* and *Flag-tSP-Cow* expression were induced along the A-P border using *ptc-Gal4* (in *ptc>myrRFP+SP-EGFP-Cow+Flag-tSP-Cow*). (C) *SP-EGFP-Cow* was induced by *ptc-Gal4* (*ptc>nlacZ+SP-EGFP-Cow*) and detected with an anti-Cow antibody. The *ptc*-expressing cells are labeled with *nlacZ* (red). The GFP fusion Cow protein, SP-EGFP-Cow (GFP, green), could be detected with an anti-Cow antibody (blue). The distribution of both signals was broader than the *ptc^+^* cells and showed a punctate signal. (D) Flag-tagged Cow with a truncated signal peptide (*Flag-tSP-Cow*) was expressed in the *ptc* (red) expression domain (in *ptc>myrRFP+Flag-tSP-Cow*). A Flag signal (green) was detected only in the Ptc domain, indicating that the signal peptide was required for its secretion. Anti-Cow antibody (blue) detected the Flag-tSP-Cow in the Ptc domain as well as the endogenous Cow present throughout the disc. In (C) and (D), the level of *ptc-Gal4*-driven Cow expression was much higher than that of endogenous Cow. (E) Extracellular GFP staining of *ptc>myrRFP+SP-EGFP-Cow* was demonstrated in the haltere; the extracellular GFP staining signal (cyan) can be detected at the Ap and Ba surfaces of the haltere. (F) The WT, *sfl^03844^/twi-GFP* and *sfl^03844^* homozygous embryo protein extracts were probed using anti-Cow antibody. Tubulin was used as the loading control. (G–L) Embryonic cuticle phenotypes of (G) wild-type (WT), (H) *cow^5Δ^*, (I) *Df(3R)BSC527/Df(3R)BSC619*, (J) *tub>Cow-miRNA-1*, (K) *tub>3xCow-dsRNA*, and (L) *wg^cx4^/+; cow^5Δ^*.

We identified the *Drosophila CG13830* gene (Figure S1A) and its human homolog Testican-2 in a gain-of-function screen. We named it *carrier of wg* (*cow*) for reasons to be described below. Cow belongs to the testican family of secreted HSPGs with a signal peptide (SP) and five conserved domains (Figure S1B) [36]. We generated a mutation, *cow^5Δ^*, with a deletion of the 3′UTR (Figure S1A) that is protein-null (Figure S1C–D). We also generated *UAS-Cow-dsRNA* and two *UAS-Cow-miRNA* constructs (Figure S1B; see Materials and Methods) that effectively reduced the Cow level (Figure S1D–E). The expression patterns of *cow* at the RNA and protein levels throughout development are presented in Figure S1. In the wing disc, Cow is detected throughout the disc and is primarily localized to the Ap surface (Figure 1A).

We next tested whether Cow can be secreted, reminiscent of its human homolog Testican-2 [37]. We generated an EGFP-Cow fusion construct (SP-EGFP-Cow) and a construct of Cow with a truncated signal peptide (Flag-tSP-Cow). Both were expressed by the *ptc-Gal4* (abbreviated as *ptc>myrRFP+SP-EGFP-Cow +Flag-tSP-Cow*) at the anterior-posterior (A-P) boundary of the wing disc (Figure 1B). Both were detectable using the anti-Cow antibody (Figure 1C–D). The GFP signal was broader than the Ptc expression region, and it appeared in puncta on both the Ap and Ba surfaces (Figure 1B–C). However, Flag-tSP-Cow was restricted to *ptc-Gal4*-expressing cells (Figure 1B and 1D). Extracellular staining using anti-GFP was performed on *ptc>myrRFP+SP-EGFP-Cow* discs to detect EGFP-Cow in the extracellular space. The GFP fluorescent signal could be detected in the *ptc*-expressing region, and the extracellular anti-GFP signal was broader than the *ptc* domain (Figure 1E). These results suggest that Cow can be secreted and dispersed away from producing cells and that this secretion requires its signal peptide. When SP-Flag-Cow was transfected into S2 cells, an anti-Flag antibody detected bands at 100 kDa and 75 kDa in the culture supernatant (Figure S1F). These results clearly demonstrated that Cow was secreted.

We then tested whether Cow is HS-modified. Cow has a predicted molecular weight of 72 kDa, and an anti-Cow antibody detected two bands at approximately 100 kDa and 75 kDa in embryos, wing discs, adults and S2 cells (Figure S1C). Both bands were lost in *cow^5Δ^* mutant embryos and were enhanced when Cow was overexpressed (Figure S1C–D). These results demonstrated the specificity of the anti-Cow antibody. The 100-kDa band was not detected in the embryonic extract of the *sfl* homozygous mutant (Figure 1F), suggesting that it was modified by HS. When the two putative GAG sites in Cow were mutated, the 100-kDa band was significantly reduced (Figure S1F), supporting the conclusion that this was the HS-modified form. In comparison, the 75-kDa band likely represented unmodified Cow. These results demonstrate that Cow, like its human homolog Testican-2, is HS-modified and that both HS-modified and unmodified Cow can be secreted.

### Genetic relationship between *cow* and *wg*

Homozygous *cow^5Δ^* mutants are mostly embryonic lethal; 50% die before cuticle formation, and 9.5% of *cow^5Δ^* embryos showed a weak denticle belt fusion phenotype (Figure 1H). Expressing *Cow-miRNA-1* or *Cow-dsRNA* in the embryo also produced similar phenotypes (Figure 1J–K). Similar embryonic denticle belt fusion phenotypes have been reported in many mutants, including mutations in the Hedgehog (Hh) and Wg signaling pathways [38]. Because Cow is a HSPG, we investigated its relationship with the morphogens Hh and Wg. When the *wg* dosage was reduced, the *cow^5Δ^* phenotype became very strong (Figure 1L), similar to the *wg^cx4^* homozygous mutant [38]. The *hh* gene is also deleted in two deficiencies with deleted *cow* (Figure S1A), but the combination of *hh* deletion and *cow* mutation showed no enhancement of the denticle fusion phenotype (Figure 1I and unpublished data). Deletion of one copy of the Hh coreceptor gene *smoothened* (*smo*) did not affect the *cow^5Δ^* embryonic phenotype (unpublished data). These results showed that *cow* has a strong genetic interaction with *wg* but not with Hh signaling.

When Cow was knocked down in the developing wing, driven by *MS1096-Gal4*, which is expressed in the entire wing pouch region in the wing disc [39], [40], ectopic chemosensory bristles developed along or near the wing margin, on both the anterior (A) and posterior (P) sides (Figure S2B–B′). In contrast, ubiquitous overexpression of Cow or human Testican-2 caused the loss of chemosensory bristles along the wing margin (Figure S2C–C′, D–D′ and E) and extra wing vein tissue (Figure S2C–D). The extra wing vein phenotype has not been reported for Wg signaling, suggesting that Cow has additional functions beyond influencing Wg. The cell fate of wing margin chemosensory bristles is determined by Wg signaling [1], [41], and ectopic chemosensory bristles were also observed upon overexpression of the Wg receptor DFz2 (Figure S2H; [28], [42]). Thus, for the wing margin chemosensory bristles, the loss of function (LOF) of *cow* produces a phenotype similar to enhanced Wg signaling, whereas gain of function (GOF) of Cow gives a phenotype similar to loss of Wg. This is in contrast to the embryonic segmentation phenotype, where loss of *cow* and *wg* caused similar denticle belt fusion phenotypes. Consistent with the lack of genetic interaction with the Hh pathway in the embryonic phenotype, neither GOF nor LOF of *cow* caused changes in the wing L3–L4 intervein area, which is regulated by Hh signaling and by Dlp [7], [43].

This genetic interaction suggested that Cow may affect Wg signaling. When *cow^5Δ^* mutant clones were generated in the wing using *ap>FLP*, 89.4% of the *cow^5Δ^* mutants died before the larval stage, and 9.3% died at the pupal stage. The escapers (1.3%) all showed a wing-loss phenotype, (Figure S2F), which is similar to the *wg^1^* phenotype. We then tested the epistatic relationship between *cow* and the Wg receptor DFz2. Expression of dominant-negative DFz2 (DFz2N), driven by *MS1096-Gal4*, caused the loss or strong reduction of wings (Figure S2G; [28]), whereas coexpression of Cow, Cow-dsRNA or Cow-miRNA with DFz2N did not affect the *MS1096>DFz2N* wing loss (Figure S2G) or the *MS1096>DFz2* ectopic bristles (Figure S2H). These results suggest that Cow affected Wg signaling at a step upstream of the receptor DFz2, possibly at Wg itself. When Cow was ubiquitously overexpressed, the expression level of *wg-lacZ* and the number of rows of *wg-lacZ^+^* cells at the D-V border in the wing disc were not changed (unpublished data). Semi-quantitative RT-PCR also showed that the *wg* RNA level in late third instar wing discs of *tub>Cow* was not changed (unpublished data). These results demonstrate that the effect of Cow on Wg signaling is not at the level of transcription, but most likely at the level of Wg protein.

### Cow affected the expression of Wg target genes in a biphasic pattern

We next investigated whether *cow* affects Wg signaling by examining the expression of Wg target genes. In the wing disc, Wg is expressed in a stripe of a few rows of cells at the D-V boundary and at the boundary of the wing pouch region [41], [44], [45]. The *wg^+^* cells at the D-V border also express Cut [41]. We examined the expression of several known Wg target genes in the wing disc: *neuralized* (*neur*), *senseless* (*sens*), and *Distalless* (*Dll*) [1], [3], [46]. Although both *neur* and *sens* are viewed as high-threshold Wg target genes, they appear to require different levels of Wg for their activation, and *sens* expression is broader than *neur* expression (see below). In particular, *neur* is only activated within and immediately adjacent to the ectopic Wg-expressing clone [1], whereas *sens* can be activated several cells away [46]. In addition, *sens* expression occurs earlier than *neur* in the same cell [33]. All of these findings suggest that *sens* responds to a lower level of Wg than *neur*. Therefore, we suggest that *neur* is a short-range target and that *sens* is an intermediate-range target.

**Figure 2:**
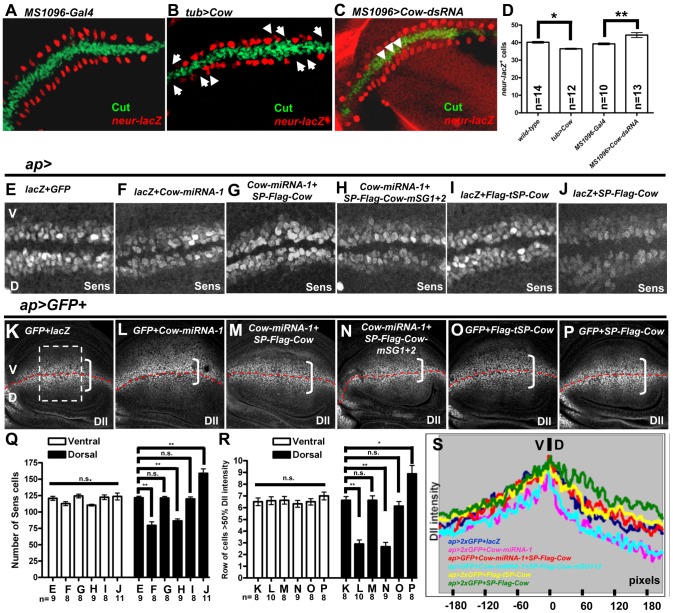
Cow affects the expression of Wg target genes in a biphasic pattern. (A–C) Cut (green) and *neur-lacZ* (red) expression along the D-V border of late third instar wing discs of (A) *MS1096-Gal4* (representing WT), (B) *tub>Cow*, and (C) *MS1096>Cow-dsRNA*. *neur-lacZ* cells form a single row on each side of the D-V border in WT. Loss of *neur-lacZ* cells in their normal location is indicated by arrows. *neur-lacZ* cells located outside of their normal location are indicated by arrowheads. (D) Summary of the numbers of *neur-lacZ* positive cells in A–C. (E–J) Sens^+^ cells (white) in the V and D sides of wing discs from the indicated genotypes. The numbers of Sens^+^ cells in (E–J) are summarized in (Q). Dll (white) expression in wing discs of the indicated genotypes. To eliminate dosage variation, the number of *UAS* constructs was equal in all groups. Data for Dll are presented as merged images of several Z sections. For Dll intensity, we chose a rectangle (shown in K) with its center localized on the intersection of the A/P and D/V axes. The results are summarized in (R) as the number of rows of cells at 50% Dll intensity and in (S) as plots of Dll intensity measured from the rectangle along the D-V axis. In this and all subsequent figures, n.s., not statistically significant, *, p<0.01, **, p<0.001.

Furthermore, *neur* expression, as shown by *neur-lacZ*, flanked the D-V border in Wg/Cut^+^ cells (Figure 2A; [1]). In *tub>Cow*, there was a loss of *neur^+^* cells in the normal domain (arrow in Figure 2B, compare with Figure 2A), accompanied by a few additional *neur^+^* cells just outside its normal domain (arrowhead in Figure 2B). When Cow was knocked down in the wing pouch area, there were extra *neur^+^* cells, and some of these extra *neur^+^* cells may be within the Cut expression domain (Figure 2C). The total number of *neur^+^* cells decreased when Cow was overexpressed and increased when Cow was knocked down (Figure 2D).

Sens is expressed in a few rows of cells along the D-V boundary (Figure 2E and S3A; [33], [46]). The number of Sens^+^ cells flanking the D-V boundary increased when either DFz2 or Cow was overexpressed (Figure S3B–C and E), whereas when Cow was knocked down, the number of Sens^+^ cells decreased (Figure S3D–E). The numbers of Sens^+^ cells were equal in the V and D sides in WT discs (Figure 2E and Q). When Cow was knocked down in the D side, driven by *ap-Gal4*, the number of Sens^+^ cells was reduced in the D compared to V side (Figure 2F and Q). This Sens^+^ reduction could be rescued by coexpression of SP-Flag-Cow (Figure 2G and Q). The Sens^+^ cells in the D side increased when SP-Flag-Cow was overexpressed (Figure 2J and Q), and the signal peptide-truncated Cow, Flag-tSP-Cow, did not show any effect on Sens^+^ cell number (Figure 2I and Q), consistent with the previous conclusion that Cow functions as a secreted protein.

Dll is expressed in a broad domain in the wing disc and is equally expressed in the D and V sides of the wing disc (Figure 2K and R–S). When Cow was knocked down in the D side, the range of Dll was reduced in this side (Figure 2L and R–S), and this reduction could be rescued by co-expression of *SP-Flag-Cow* (Figure 2M and R–S). Overexpression of Cow in the D side caused expansion of the Dll range (Figure 2P and R–S), whereas expression of Flag-tSP-Cow did not affect the Dll range (Figure 2O and R–S), confirming that Cow acts as a secreted form.

In summary, *cow* showed a biphasic effect on Wg target genes in the wing disc. For the short-range target *neur*, *cow* had the opposite effect of Wg signaling. For the intermediate-range target Sens and the long-range target Dll, *cow* had the same effect as Wg. Similar biphasic effects have been reported for Dlp [7], [16], [17], [18], [19], [20].

### HS-modified Cow promoted exWg movement and stability

**Figure 3:**
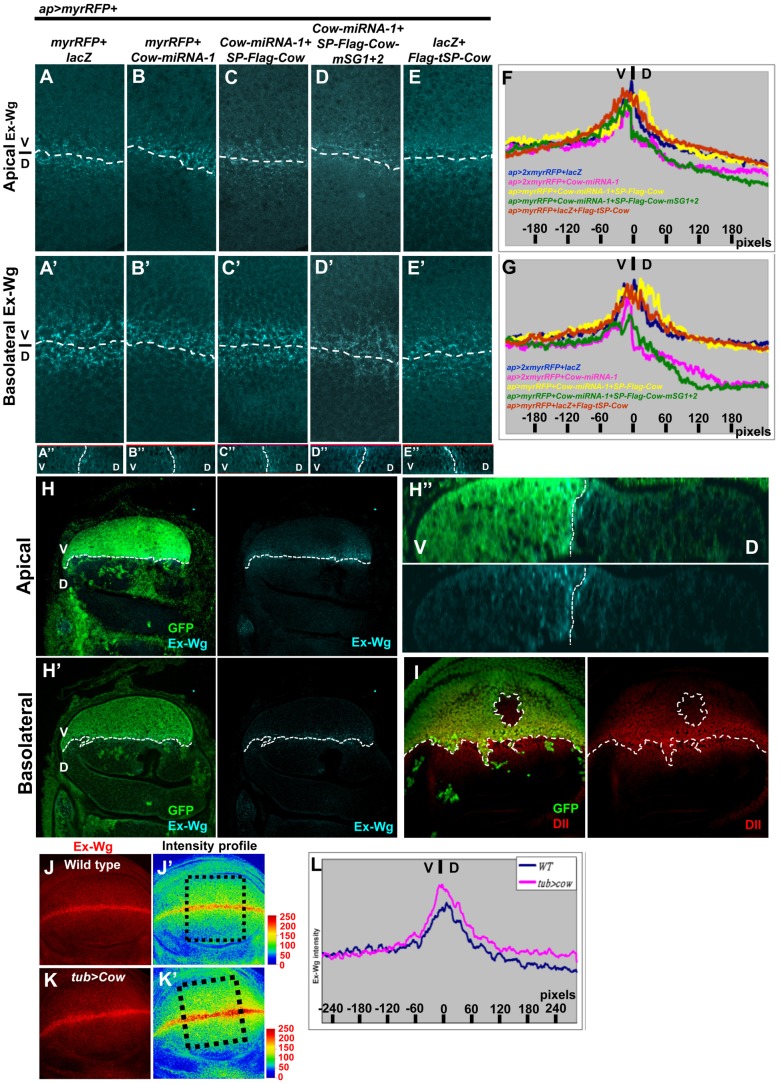
Cow promotes the extracellular distribution of Wg. (A–E) Staining of exWg (cyan) in wing discs of the indicated genotypes. (A″, B″, C″, D″ and E″) Z sections of each combination are shown. The number of *UAS* constructs was equalized to eliminate dosage differences. The exWg intensity plots (see Materials and Methods) for each condition at the Ap (F) and Ba surfaces (G). The exWg distribution (H–H″) and Dll range (I) were examined in the *cow^5Δ^* mutant clone. Staining of exWg (red) in (J) WT and (K) *tub>Cow*. The intensity of the exWg signal in J and K is color-coded in J′ and K′, respectively, and intensity plots are shown in (L).

The apparent contrast between wing and embryo phenotypes and the biphasic activity of target genes can be explained by a simple unifying model in which Cow promotes Wg movement, thereby reducing the Wg level near its source and broadening its distribution. We used an immunostaining protocol to measure the extracellular distribution of Wg produced from the D-V boundary in the wing disc [5] and tested whether Wg distribution is promoted by Cow. In the control wing disc, exWg showed a similar distribution in the D and V sides, in both Ap and Ba regions (Figure 3A–A″ and F–G). When Cow was knocked down in the D side, the exWg range in D was reduced in both the Ap and Ba domains (Figure 3B–B″ and F–G). This reduction in the range of exWg could be rescued by co-expressing *SP-Flag-Cow* (Figure 3C–C″ and F–G), indicating that the effect was specific to Cow and ruling out off-target effects of the *Cow-miRNA*. We also generated a *cow^5Δ^* mutant clone in D using *ap>FLP*, in which the exWg level is significantly reduced in both the Ap and Ba domains (Figure 3H–H″), and the Dll range was also reduced (Figure 3I).

We then tested the requirement for HS modification of Cow. Cow with mutations in the putative GAG sites failed to rescue the reduction of range of exWg (Figure 3D–D″ and F–G) and Sens and Dll (Figure 2H, N, and Q–S) caused by *Cow-miRNA-1*. These results suggest that the HS modification is required for Cow to promote Wg movement.

When Cow was ubiquitously overexpressed, the exWg distribution along the D-V axis was broader than that in WT cells (Figure 3J–K), which were stained and imaged in parallel under the same conditions. This difference was best shown by color-coding (compare Figure 3J′ and K′) and intensity plots (Figure 3L). The Flag-tSP-Cow did not change the range of exWg (Figure 3E–E″ and F–G), suggesting that this effect was mediated by secreted Cow.

One possible explanation for this increased range of distribution is that Cow may stabilize exWg. Therefore, we measured the total exWg signal over the entire wing disc by confocal microscopy. *tub>Cow* wing discs were 5.8% smaller than WT discs, and when normalized for wing disc size, *tub>Cow* produced a 12.6% increase in exWg (n = 16 for WT and n = 12 for *tub>Cow*). This result suggested that Cow could increase the stability of exWg because Cow did not change *wg* transcription and acted as a secreted form. We also tested whether Cow stabilized Wg in the extracellular environment. Secreted HA-Wg from transfected S2 cells was added to S2 cells that were transfected with *Cow-dsRNA* vectors (Figure S4A–C). The level of HA-Wg in the supernatant was decreased by 47% in the *Cow-dsRNA*-transfected S2 cells after 48 h in culture, whereas this level was decreased by only 7% in control S2 cells (Figure S4A and C). These results strongly support a model in which Cow protein is required for exWg stability.

### HS-modified Cow can bind to Wg extracellularly

**Figure 4:**
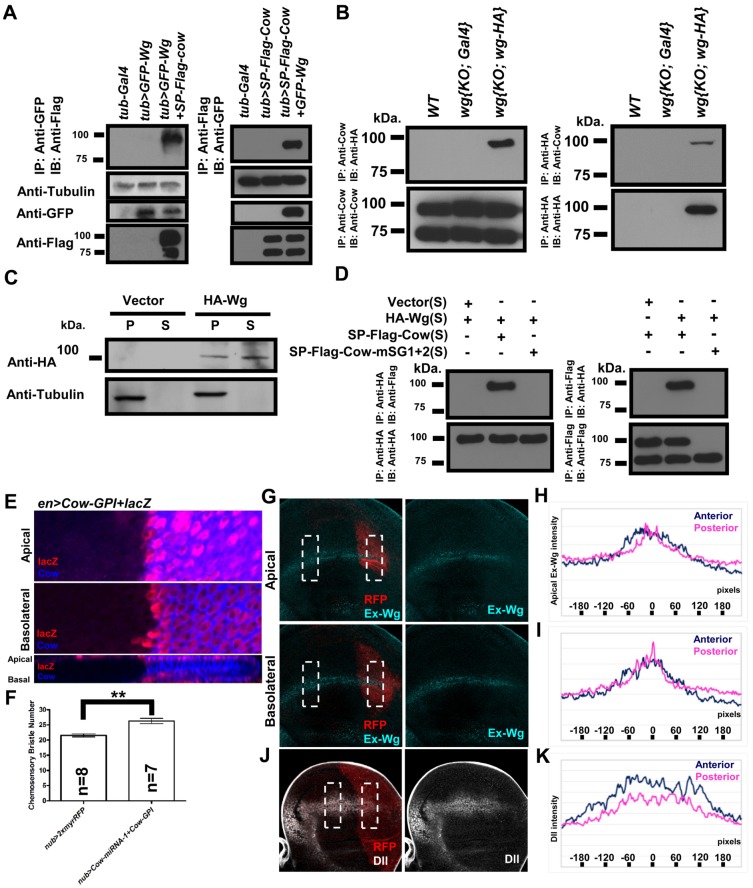
HS-modified Cow can bind to Wg. (A) The embryo extracts were immunoprecipitated with anti-GFP and then probed with anti-Flag on western blot. When GFP-Wg and SP-Flag-Cow were coexpressed (in *tub>GFP-Wg+SP-Flag-Cow*), a broad band at approximately 100 kDa was detected. This band was not detected in extracts from WT (*tub-Gal4*) or *tub>GFP-Wg* embryos. In the reverse experiment, embryo extracts were immunoprecipitated with anti-Flag and then stained with anti-GFP. The GFP signal was detected in *tub>SP-Flag-Cow+GFP-Wg* but not in *tub-Gal4* or *tub>SP-Flag-Cow*. (B) The larval extracts of *WT*, *wg{KO; Gal4}* and *wg{KO; wg-HA}* were immunoprecipitated with anti-HA or anti-Cow and then probed with anti-Cow and anti-HA antibodies, respectively. (C) S2 cells were separately co-transfected with *actin-Gal4* plasmid and empty *UAS* vector, or *UAS-HA-Wg*. After 48 h, the cell pellet (P) and supernatant (S) were probed using western blotting. HA-Wg could be detected by anti-HA as a band at approximately 100 kDa, consistent with a previous report on HA-Wg [22]. (D) HA-Wg, SP-Flag-Cow and SP-Flag-Cow-mSG1+2 were separately expressed in S2 cells, and then the culture supernatants were mixed in different combinations, immunoprecipitated and probed in western blots. When anti-HA was used to immunoprecipitate, anti-Flag detected Flag-Cow at approximately 100 kDa but did not detect in Flag-Cow-mSG1+2. These results indicate that only the HS-modified Cow can interact stably with Wg. The Anti-HA blot showed that HA-Wg was expressed at similar levels in the three samples. Using anti-Flag for immunoprecipitation, anti-HA detected HA-Wg as the expected 100-kDa band. Anti-Flag blotting showed that both the 75-kDa and 100-kDa bands of Cow were present in the supernatant. (E) Cow-GPI was expressed in the P compartment of the wing disc (in *en>Cow-GPI+lacZ*). Anti-Cow (blue) detected Cow in the P compartment. The signal at the Ap surface was much stronger than that at the Ba surface. The induced expression level was also much higher than the endogenous Cow level, which was below the detection threshold under these conditions. There was no Cow signal outside the cells where it was expressed (marked by *lacZ*, red), indicating that the GPI-anchored Cow remained within the cells where it was produced. (F) Chemosensory bristle numbers from *nub-Gal4>2xmyrRFP* and *nub>Cow-miRNA-1+Cow-GPI* are summarized. Ectopic expression of Cow-GPI increased the chemosensory bristle numbers on the adult wing margin. With expression of Cow-GPI (in *tub>Cow-GPI*), the wing margin chemosensory bristles increased from 22.60±0.31 (*WT*) to 24.15±0.48 (*tub>Cow-GPI*), representing a 6.9% increase. When Cow-GPI was expressed in combination with Cow knockdown (*nub>Cow-miRNA-1+Cow-GPI*), the chemosensory bristle number increased from 21.57±0.48 in controls (*nub>2xmyrRFP*) to 26.29±0.84 (*nub>Cow-miRNA-1+Cow-GPI*), representing a 21.9% increase. (G) Most of the *hh>myrRFP+Cow-miRNA-1+Cow-GPI* individuals died before the larval stage, and the remaining 3.8% escapers were dissected for exWg staining. Ap and Ba exWg staining of *hh>myrRFP+Cow-miRNA-1+Cow-GPI*. A box of 120×240 pixels was chosen at a symmetrical position in the A and P sides of the Ap and Ba surfaces. There are 120 single-pixel lines perpendicular to the D-V border. The intensity values of ten adjacent lines were averaged to produce 12 groups from one disc. The results from two discs were then averaged to obtain the intensity plots (H and I). The results from the P side (red) and A side (blue) were compared. Compared with the A side, the exWg in the P side showed a narrower distribution. (J) The level and range of distribution of Dll were similarly analyzed in *hh>myrRFP+Cow-miRNA-1+Cow-GPI*. The P side showed a narrower range of expression of Dll than the A side. (K) The Dll plot of *hh>myrRFP+Cow-miRNA-1+Cow-GPI*. For (G), **, p<0.001.

We then tested whether Cow can physically interact with Wg by coexpressing epitope-tagged forms of Cow and Wg (*tub>GFP-Wg+SP-Flag-Cow*). Coimmunoprecipitation from embryo extract showed that HS-modified Cow interacted with Wg (Figure 4A). We further tested whether Cow and Wg could form a protein complex at endogenous levels. In the *wg{KO; wg-HA}*, the *wg* was replaced with *wg-HA* in the endogenous genomic locus [29], [30]. Coimmunoprecipitation from larval extract again showed that only the HS-modified Cow formed a protein complex with Wg-HA (Figure 4B).

When HA-Wg and SP-Flag-Cow were separately expressed in S2 cells and the supernatants (Figure 4C) were mixed, coimmunoprecipitation showed that Cow and Wg interacted after their secretion, and the interaction with Wg involved only the HS-modified form of Cow. This result was further supported by the finding that the interaction with Wg was lost when the GAG sites in Cow were mutated (Figure 4D). The same extracellular binding assay also showed that human Testican-2 could bind Wnt5a extracellularly (Figure S4D–G), suggesting that the Testicans may have an evolutionarily conserved role in regulating Wnt signaling.

We then tested whether the binding between Cow and Wg occurs in vivo. We generated a GPI-anchored Cow (Cow-GPI) to test whether membrane-anchored Cow would restrict exWg movement. Expression of Cow-GPI in the P domain showed that Cow-GPI was indeed restricted to P in both the Ap and Ba surface (Figure 4E) and that it caused an increase in chemosensory bristles (Figure 4F). Although the *en>Cow-GPI* expression level was much higher than that of endogenous Cow (Figure 4E), endogenous Cow was knocked down to observe the full effect of Cow-GPI on Wg. The exWg range became narrower in the P region compared to the A region, whereas the exWg level at the D-V border became higher at the Ap (Figure 4G–H) and Ba (Figure 4G and I) surfaces. Similarly, the Dll range became narrower in the P region (Figure 4J–K). When *Cow-GPI* expression was combined with Cow knockdown in the wing pouch, there was a significant increase in chemosensory bristles along the wing margin (Figure 4F). These results are consistent with the expectation that membrane-anchored Cow would bind Wg and restrict its movement, thereby reducing its range but retaining more Wg at its source, thus providing in vivo evidence that Cow can bind to Wg and affect its distribution.

### Cow enhanced the rate of extracellular movement of Wg

**Figure 5:**
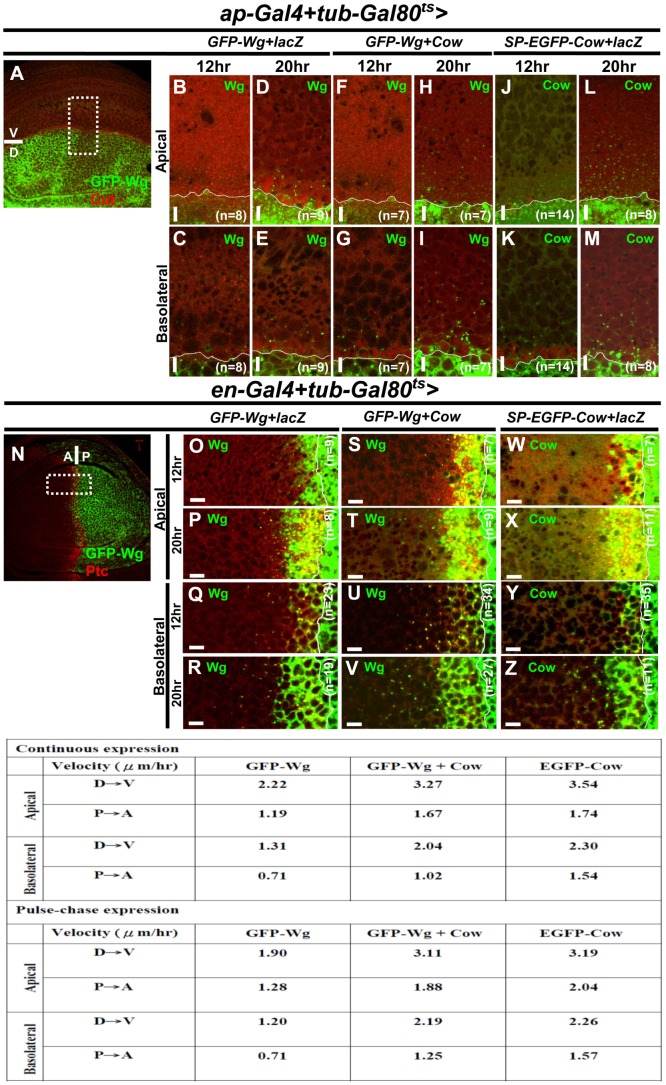
Cow enhances the rate of extracellular movement of Wg. For continuous expression, the *ap-Gal4*+*tub-Gal80^ts^* (A-M) or *en-Gal4*+*tub-Gal80^ts^* (N-Z) larvae were shifted from 17°C to 30°C to induce UAS-transgene expression. The boxed areas in A and N were enlarged and shown in B-M and O-Z. The entire wing disc was divided into 10 equal sectors parallel to the D-V axis in *ap-Gal4*+*tub-Gal80^ts^* and to the A-P axis in *en-Gal4*+*tub-Gal80^ts^*. The distances between the farthest signal puncta (arrowhead) and the D-V or A-P border for each sector were averaged to obtain the travel distance. The results from multiple discs were then averaged for each time point. The differences in distance traveled from 12 h to 20 h were used to calculate the velocity of movement and are summarized in the table. For pulse-chase expression, the *ap-Gal4+tub-Gal80^ts^* and *en-Gal4+tub-Gal80^ts^* flies were shifted to 30°C for 12 h to induce GFP-Wg expression and then shifted back to 17°C to turn off GFP-Wg expression. The GFP-Wg movement during the 8 h following the end of the induction was monitored. The calculated velocities are summarized in the table.

One possible mechanism by which Cow might promote Wg movement is through binding to Wg and enhancing its rate of movement. We transiently expressed GFP-Wg in the presence or absence of coexpressed Cow, and we followed the movement of Wg over time. We combined *ap-Gal4* or *en-Gal4* with *tub-Gal80^ts^* and shifted the temperature from 17°C to 30°C during the third instar to induce GFP or GFP-Wg expression. The *ap-Gal4* and *en-Gal4* allowed us to observe the movement of Wg and Cow proteins along the D-V axis and A-P axis, respectively (Figure 5A–E, N-R, and S5A). The distances of GFP-Wg puncta signals from the D-V and A-P borders at 12 and 20 h after the temperature shift (Figure 5B–E, O–R and Table) were used to estimate the apparent rate of movement of GFP-Wg during this interval. EGFP-Cow moved 1.46- to 2.17-fold faster than GFP-Wg (Figure 5Table), and when Cow was co-expressed, the apparent mobility of GFP-Wg increased by 47% at Ap and 56% at Ba surfaces along the D-V axis and by 40% at the Ap surface and 44% at the Ba surface along the A-P axis (Figure 5, F–I, S–V and Table). After a transient 12-h induction, Wg showed similar mobility (Figure 5, Table). Thus, we propose that by binding to Wg, Cow serves as a carrier to enhance the speed of Wg movement; as a result of this role, we named the protein Carrier of Wg (Cow).

Strigini and Cohen (2000) used a different method to measure the rate of Wg movement; these authors shifted *shi^ts^* mutants to a non-permissive temperature to deplete exWg and internalized Wg in the receiving cells. Shifting back to a permissive temperature allowed the reinitiation of Wg secretion and the estimation of its rate of movement. We modified this approach by expressing Shi^ts^ in the wing disc, with or without Cow knockdown, and applied the same temperature-shift regime. The Wg distribution was examined immediately or 60 min after shifting back to the permissive temperature. The rates of Wg movement in *nub>Shi^ts^+lacZ* were 5.04 µm/h at the Ap surface and 10.15 µm/h at the Ba surface during this interval (Figure S5B–E and J). With Cow knockdown, the Wg rates decreased to 0.93 µm/h at the Ap surface and 2.12 µm/h at the Ba surface (Figure S5F–J). This result showed that Cow is required for enhancing the rate of Wg movement. In addition, the Wg level was lower in the *nub>Shi^ts^+Cow-miRNA-1* discs than in WT, consistent with the above in vitro result (Figure 3 and S4A–C) showing that Cow also stabilized Wg.

**Figure 6:**
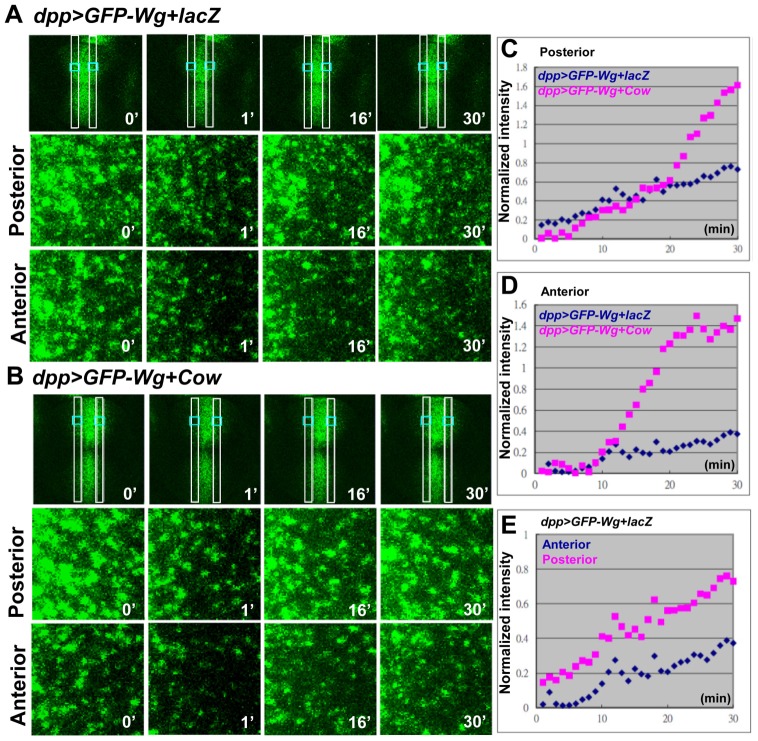
Cow enhances GFP-Wg recovery in FRAP. GFP-Wg was ectopically expressed in the *dpp* domain without (*dpp>GFP-Wg+lacZ*) (A) or with Cow (*dpp>GFP-Wg+Cow*) (B) coexpression. GFP-Wg recovery was analyzed in the A and P compartments using FRAP time-lapse images. The FRAP images were recorded before photobleaching (0 min) and after photobleaching (1, 16 and 30 min) at A and P. Normalized intensity of GFP-Wg recovery from *dpp>GFP-Wg+lacZ* and *dpp>GFP-Wg+Cow* at P (C) and A (D). The recovery in *dpp>GFP-Wg+Cow* overshoots at 20–30 min and returns to the original intensity at approximately 1–2 h. (E) The recovery curve of GFP-Wg showed faster recovery in P than in A.

To further investigate whether Cow enhanced the movement of Wg, FRAP was used to monitor the kinetics of GFP-Wg spreading [6]. At both the A and P regions, the GFP-Wg recovery was faster in *dpp>GFP-Wg+Cow* than in *dpp>GFP-Wg+lacZ* (Figure 6A–D), strongly confirming that Cow can enhance GFP-Wg movement. Furthermore, GFP-Wg showed more rapid recovery in the P region than in the A region (Figure 6E). Although Kicheva et al. (2007) [6] calculated Wg mobility based on a non-directional model, they performed FRAP only in the P compartment. Our experimental results showed that Wg mobility varied between directions, suggesting an additional level of regulation.

Next, we asked whether this enhancement of Wg speed by Cow is relevant during development. To this end, we monitored the Wg distribution, Dll pattern and wing pouch size in Cow GOF and LOF mutants during wing disc development. During the period of 84–112 h after egg laying (AEL), the disc growth resulted in the wing pouch margin moving away from the D/V boundary at 0.98 µm/h for the D margin and 1.50 µm/h for the V margin (Figure S5K–P). These results were within the same range as our estimate of Wg movement. Thus, the enhancement of Wg mobility by Cow matches the requirement for the establishment of a Wg gradient during this short developmental time period.

### Cow promoted Wg Ap transport independent of endocytosis

**Figure 7:**
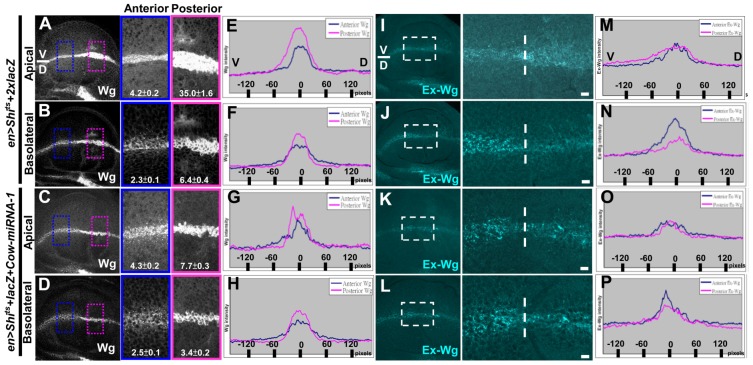
Cow promotes the Ap transport of Wg. (A–B) *en>Shi^ts^+2xlacZ* (n = 14) and (C–D) *en>Shi^ts^+lacZ+Cow-miRNA-1* (n = 11) larvae raised at 17°C were shifted to 32°C for 6 h at late third instar and were examined immediately using conventional Wg staining. Because the signal intensity varies among discs, the A compartment served as an internal control for each disc. For intensity plots, the intensity in each disc was normalized to the background and maximum signal intensity. (E–H) The Wg signal intensity in each boxed region (A–D) was transformed into an intensity plot. P, red; A, blue. (I–J) The exWg staining in the Ap (I) and Ba (J) surfaces of *en>Shi^ts^+2xlacZ* (n = 21). (K–L) The exWg staining in the Ap (K) and Ba (L) surfaces of *en>Shi^ts^+lacZ+Cow-miRNA-1* (n = 13). The expansion of Ap exWg in (I) was blocked in (K) (100%). The Ba exWg was similar in (J) and (L) (100%).

We next tested whether Cow-mediated Wg transport occurs at the Ap or Ba surface, as well as whether it is dependent on endocytosis. Endocytosis was blocked in the P region by expressing Shi^ts^ and shifting the temperature from 17°C to 32°C for 6 h. Because there is variation between discs, the A compartment in the same disc can be used as an internal control for quantitative comparison with the P compartment. Total Wg accumulation was higher in the P region (8.3X in Ap, 2.8X in Ba) than A region, and its distribution was also wider (Figure 7A–B and E–F). The expression of exWg in the P region was higher and broader at the Ap surface but lower and narrower at the Ba surface (Figure 7I–J and M–N). These results are consistent with Ap transport being independent of endocytosis and with transcytosis being responsible for the movement of Wg to the Ba surface [5], [7], [9]. Because Shi is also required for Wg secretion, the effect may also be due to a block in Wg secretion. However, when Wg secretion was blocked in *evi^2^* mutant clones, exWg was strongly reduced [47]. Thus, the wider distribution of exWg and Ap accumulation was not due to a block of secretion by Shi^ts^. Moreover, the higher level of Wg in the endocytic-deficient P compartment is consistent with endocytic Wg being targeted for degradation [5].

When Cow was knocked down in the P region together with endocytosis blockage, the accumulation and expansion of Ap Wg were significantly reduced (Figure 7C and G), and a similar effect was found for exWg (Figure 7K and O). The range of Wg was broader in *en>Shi^ts^* cells (P>A in Figure 7A) but was reduced in *en>Shi^ts^+Cow-miRNA-1* cells (A>P in Figure 7C), and the range of exWg was wider in *en>Shi^ts^* cells (P>A in Figure 7I) and reduced in *en>Shi^ts^+Cow-miRNA-1* cells (A>P in Figure 7K). These results suggest that the Ap transport of Wg depends on Cow. The Ba distribution of Wg was similar to that observed in *en>Shi^ts^+2xlacZ* (Figure 7D, H, L and P), suggesting either that Cow does not play a major role in the transcytosis of Wg through the Ba surface or that Cow activity is dependent on Shi function. Furthermore, the accumulation of Wg was reduced on both the Ap and Ba surfaces (Figure 7C–D and G–H), supporting the idea that Cow stabilizes exWg. Together, these results show that Cow is required primarily for exWg transport at the Ap surface and is not dependent on endocytosis. At 84 h AEL, Wg appeared primarily at the Ap surface around the D/V border (unpublished data), supporting the importance of Wg Ap transport for establishment of the Wg gradient.

## Discussion

### Cow serves as a carrier of Wg to promote Wg extracellular movement

In this study, we showed that Carrier of Wg (Cow) is a secreted HSPG that can physically interact with Wg (Figure 4). The binding to Wg is dependent on the HS modification on Cow, and it can occur after both proteins are secreted (Figure 4). We also measured the apparent rates of Cow and Wg movement (Figure 5), and our results showed that (a) Cow moved faster than Wg, (b) overexpression of Cow enhanced the rate of Wg movement, and (c) knockdown of Cow reduced the rate of Wg movement. Thus, we suggest that Cow serves as a carrier of Wg to enhance the rate of its extracellular movement. This enhancement of Wg movement by Cow is important for the establishment of the Wg gradient during development. Moreover, the role of Cow as a carrier for a morphogen is unique among HSPGs in that Cow is a secreted HSPG, whereas the previously studied syndecans and glypicans are membrane-bound.

The measurements of Wg and Cow mobility were performed using endogenous Wg and Cow. Therefore, the true mobility of Wg and Cow, without the presence of the other, has not been determined. Because the *cow* mutant phenotype is dominantly affected by reducing the *wg* dosage (Figure 1) and because overexpression affected Wg signaling (Figure 2, 3 and S3), we expect that neither is present in large excess over the other. The *ptc-Gal4*-driven expression of Cow was also much higher than the endogenous level of Cow (Figure 1C–D). Therefore, we expect that EGFP-Cow would be expressed much more highly than endogenous Wg, and this measurement represents the mobility of free EGFP-Cow. The large excess of overexpressed Cow likely enabled the measurement of its lateral mobility independent of endocytosis.

We used two methods to measure the mobility of Wg. The first was to use Gal4/Gal80^ts^ and temperature shifting to transiently induce Wg expression and then measure Wg distribution within an 8-h period. The estimated rates were 2.22 µm/h and 1.31 µm/h at the Ap and Ba surfaces, respectively, for the D-V axis. The second is a modification of the method used by Strigini and Cohen (2000) [5]. The estimated rate of Wg movement was 5.04 µm/h at the Ap surface and 10.15 µm/h at the Ba surface. In contrast to the first method, which addresses newly synthesized Wg, the second method addresses the release of intracellularly accumulated Wg, which is therefore driven by a higher concentration gradient and thus shows higher mobility. However, the second method indicated much slower rates than the calculated rate of 50 µm/30 min reported by Strigini and Cohen (2000) [5]. One important difference is that Strigini and Cohen (2000) used *shi^ts^* mutant discs [5], whereas we expressed Shi^ts^ in the wing disc using *nub-Gal4*. Moreover, this difference was likely caused by the incomplete blocking of Wg secretion by the dominant-negative Shi^ts^, as evidenced by the exWg distribution in *en>Shi^ts^*.

Several extracellular molecules have been reported to influence Wg trafficking, including secreted Wingless-interacting molecule (Swim) and Lipophorin [48], [49], which are involved in Wg long-range travelling; exosomes [50], which do not affect Wg gradient formation [51]; and Secreted Frizzled-Related Proteins (SFRPs) [52], [53], which have no homolog in *Drosophila*. Our study shows that Cow is required for the short-range transport of Wg. Because Wg is lipid-modified [54], its diffusion may be hindered by hydrophobic interactions with cell membranes. Its interaction with Cow may also help to reduce its interaction with the cell membrane, thereby accelerating its movement.

We note that because Cow is a secreted protein and appears uniformly in the wing disc, it cannot provide directionality to Wg transport. Instead, it simply enhances the mobility of Wg and allows the Wg gradient to be established more quickly.

### The role of Cow in the formation of the exWg gradient

**Figure 8:**
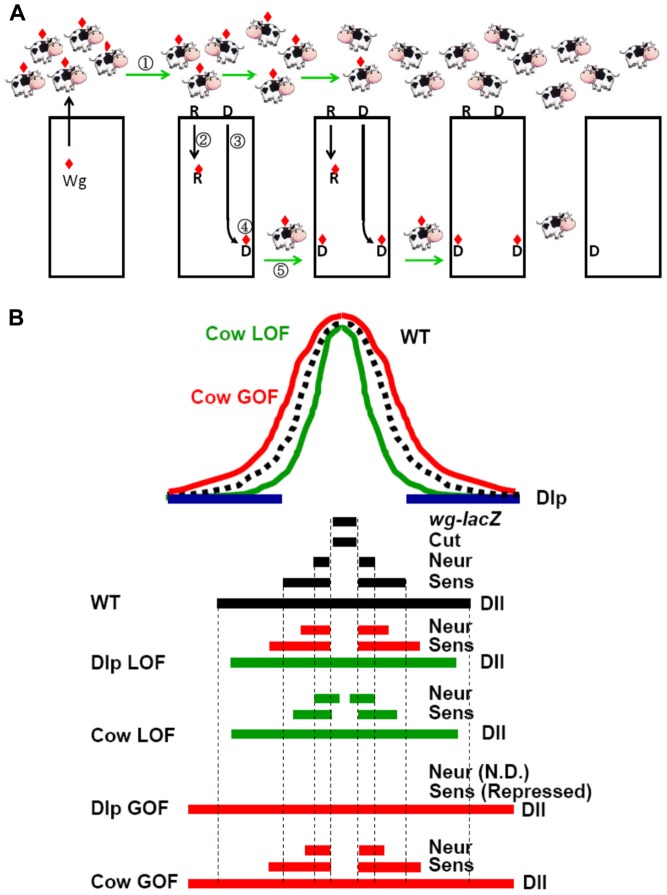
Roles of Cow in formation of the Wg gradient. (A) Our model for shaping the Wg gradient. Wg (red diamond) is produced by the D-V border cells (leftmost cell) and is secreted at the Ap side. This Wg can then be spread by several pathways: (1) ExWg transport on the Ap surface is independent of Dlp and endocytosis. Our results suggest that Cow binds to Wg and enhances its mobility on the Ap surface. (2) Wg can be internalized by receptor (**R**)-mediated endocytosis, which targets it for lysosomal degradation [15]. (3) Wg can also be internalized by Dlp (**D**)-mediated transcytosis, an intracellular process that translocates Wg from the Ap to the Ba surface. Cow does not play an essential role in the two Wg internalization processes because Wg internalization can still occur when Cow is knocked down. (4) Wg is then released to the outside of the cell, presumably by exocytosis at the Ba surface. (5) The Ba exWg movement across the intercellular gap is presumably facilitated by binding to Cow. In summary, Cow participates in steps 1 and 5 described above to facilitate the extracellular distribution of Wg. (B) Summary of Cow and Dlp LOF and GOF effects on Wg distribution and Wg target gene expression. The expression ranges of Dlp, *wg-lacZ*, Cut, *neur-lacZ*, Sens and Dll along the D-V axis are shown. Red, expansion of target gene; green, reduction of target gene.

We propose that Cow plays two distinct roles in the formation of the exWg gradient (Figure 8A). (1) Cow is responsible for the Ap transport of exWg. This process is independent of endocytosis and is especially important because it is responsible for moving Wg out from its producing cells, which express only low levels of DFz2 and Dlp. (2) Cow is responsible for the intercellular transfer of Wg at the Ba surface. Because Cow is diffusible, it is expected to be more efficient to carry Cow over the intercellular space than to exchange Wg between membrane-bound Dlp or other receptors on adjacent cells. In addition, Cow also slightly increased exWg stability, perhaps by binding to Wg, or by diverting Wg away from endocytosis and degradation.

Because Wg can be bound by its receptor DFz2, by Dlp and Dally, and by Cow, these factors may compete for binding to Wg. It has also been proposed that the relative levels of Wg, DFz2 and Dlp can affect the morphogen activity gradient [17]. Our study adds another potential binding partner to this process. The relative levels of Wg, DFz2, Dlp and Cow likely determine not only the shape of the Wg gradient but also its relative distribution on the Ap versus Ba surfaces.

The biphasic activity of Cow can be explained by its effect on Wg mobility (Figure 8B). Cow knockdown reduced Wg mobility, causing Wg to spread less and accumulate near the Wg-producing region. For short-range targets, the effect was similar to Wg GOF, whereas for intermediate- and long-range targets, the effect was similar to Wg LOF. This model can also explain the apparent contrast between the wing and embryo phenotypes in Cow knockdown. In the embryo, Wg specifies the naked cuticle fate over 5 rows of cells in the anterior segments. Therefore, the naked cuticle fate can be viewed as a long-range target of Wg, in which Wg LOF loses the naked cuticle fate and produces a denticle fusion phenotype. Cow knockdown also produces a phenotype similar to Wg LOF. In the wing, the chemosensory bristles at the wing margin are controlled by the short-range target *neur* [55], and Cow knockdown caused an increase in chemosensory bristles, similar to the effect of Wg GOF.

### Gradient vs. cellular memory

Recently, it was shown that replacement of endogenous Wg with a membrane-tethered Wg is sufficient for wing development with normal patterning [30]. It has also been suggested that early Wg expression is coupled to cellular memory of target gene expression and that the spreading of Wg is therefore dispensable for patterning. However, this hypothesis does not readily explain how different Wg target genes are expressed at different ranges from the Wg source, which can be explained by the Wg gradient model and is supported by previous studies [1], [5], [7], [9], [14], [15], [16], [17], [18], [19], [20], [21], [22], [42]. In addition, we found that Dll expression is activated only after 84 h AEL (Figure S5), which is past the early Wg expression phase suggested for cellular memory [30]. The contradiction between the two modes of Wg patterning mechanisms requires further study for clarification [56].

### Conserved function and medical implication

The mammalian testicans can regulate neurite outgrowth [57] and proteases activity [58], [59], [60], [61], [62]. However, the role of testicans in regulating signaling pathways has not been studied. Our study on the fly testican Cow is the first to demonstrate a role for the testican family in morphogen signaling as a diffusible HSPG. In addition, we showed that human Testican-2 could bind to Wnt5a extracellularly, suggesting that the testican family may have a general role in regulating Wnt distribution and thus Wnt signaling.

Misregulation of Wnt signaling is well known to contribute to human diseases, including cancer [63]. Accordingly, components of the Wnt signaling pathway have been developed as therapeutic targets for cancer [64]. The Reggie protein, which affects Wnt secretion and spreading, is also associated with various types of cancer [65]. Our original identification of Cow was the result of an overexpression screen of genes with elevated expression in human hepatocellular carcinoma [66]; thus, Cow may be involved in oncogenesis. Moreover, our finding of the novel and conserved role of the testican protein family in binding to Wnt ligands may reveal their involvement in human diseases.

## Supporting Information

Figure S1 **Characterization of** ***cow*** **.** (A) The *cow* locus and deletion mutants are shown. *Mi{ET1}CG13830^MB00767^* is a Minos transposon inserted into the 3′-UTR of *cow*. Using imprecise excision, we generated a mutation, *cow^5Δ^*, which has a deletion beginning at 396 bp downstream of the *cow* open reading frame (ORF) and extending 9,119 bp downstream. The coding region of *cow* was not affected, but part of the 3′-UTR, including two putative polyadenylation signals, was deleted. RT-PCR for the *cow* coding region showed that the *cow* transcript was present in the *cow^5Δ^* mutant embryo at levels comparable to that in WT (unpublished data). However, Cow protein, as detected in western blot using an anti-Cow antibody we generated, was undetectable in *cow^5Δ^* embryo extracts (Figure S1C–D). Thus, *cow^5Δ^* is a protein null mutant. The downstream *CG17111* gene was also deleted in *cow^5Δ^*. RT-PCR confirmed that *cow^5Δ^* embryos contained no *CG17111* transcript, whereas the *CG6697* further downstream was not affected (unpublished data). *cow^5Δ^* homozygotes are lethal; 65.5% die at the embryonic stage and the rest die at the first instar. A combination of two deficiencies, *Df(3R)BSC527/Df(3R)BSC619*, which deleted the entire coding region of *cow*, and *cow^5Δ^/Df(3R)Exel6193* also showed similar early lethality and denticle belt fusion phenotypes (Figure 1I and unpublished data). These results suggest that *cow^5Δ^* is a functionally null mutation. When Cow was knocked down in *tub>Cow-miRNA-1* (Figure 1J) and *tub>3xCow-dsRNA* (Figure 1K), the cuticle phenotype was much stronger than that of *cow^5Δ^*, although the protein level was only reduced in these knockdowns (Figure S1D). It is possible that the knockdown was not complete in the early stages, as shown by the residual Cow level, thus allowing the embryos to develop past the early phase of *cow* requirement and allowing the full strength of the *cow* phenotype to be observed. In the *cow^5Δ^*, *Df(3R)BSC527/Df(3R)BSC619* and *cow^5Δ^/Df(3R)Exel6193* mutants, only the weaker phenotype was observed. Expressing *cow*, using *UAS-Cow* driven by *da-Gal4* (abbreviated as *da>Cow*), in the *cow^5Δ^* mutant nearly completely rescued the lethality, and the adults showed no apparent phenotype. These results demonstrate that *cow^5Δ^* lethality was due to the loss of Cow. The embryonic lethality of *tub>Cow-miRNA-1* could also be rescued to adulthood by coexpression of a *cow* transgene without the 5′-UTR target for *Cow-miRNA-1* (Figure S1B). The efficient rescue excluded off-target effects of the miRNA. In situ hybridization showed that *cow* was expressed relatively uniformly in the early embryo beginning at the cellular blastoderm and that it developed a segmentally repeated pattern at stage 13 (unpublished data). There was also evidence of maternal contribution, as *cow* embryos devoid of maternal contribution generated by germline clones died before cellularization (unpublished data). Cow expression was undetectable in *Df(3R)BSC527* (unpublished data), which deleted nearly the entire *cow* gene. Cow is expressed throughout the imaginal discs (unpublished data). RT-PCR showed that the *cow* transcript level is highest in the embryo, low in larva, and increased in pupa and adult (unpublished data). RNA-Seq and RNA tiling microarray data [67] show that the *cow* transcript is expressed at low levels in early embryonic stages (0–12 h), increases after 12 h, peaks at 16–18 h and is maintained until the end of embryonic stage. It then gradually decreases during larval stages and increases to high levels during pupal and adult stages. These results are consistent with our findings. Cow is also expressed at high levels in the larval and adult CNS [68]. (B) The Cow protein contains five domains conserved in the testican family: signal peptide (SP, amino acids 1–35), N region (N, 36–104), follistatin-like domain (FS, 168–274), extracellular Ca^2+^ binding EF-hand motif (EC, 428–531), thyroglobulin-like domain (TY, 535–594) with a CWCV motif, and C region (602–625). There are two GAG attachment sites in the FS domain. (C) In western blot, an anti-Cow antibody detected two bands at approximately 100 kDa and 75 kDa, respectively, in WT (*tub-Gal4*) embryos (E), wing disc (W), adults (A) and *act>GFP* S2 cells. Both bands were enhanced when SP-Flag-Cow was expressed in flies (*tub>SP-Flag-Cow*) or in S2 cells (*act>SP-Flag-Cow*). *cow^5Δ^* homozygous embryos showed no detectable Cow protein. Tubulin served as a loading control. (D) Knockdown of Cow by the ubiquitous expression of either *Cow-miRNA-1* or *Cow-dsRNA* reduced the level of Cow protein. Tubulin served as a loading control. *tub>Cow-miRNA-1* reduced the level of Cow, but *tub>3xCow-dsRNA* produced a much stronger reduction of the Cow level. These mutants were lethal, with over 90% death at the embryo stage and the remaining individuals dying at the first instar. The embryo cuticle phenotype is similar to that of the *cow* mutant. (E) When *Cow* was knocked down by *ap-Gal4* (*ap>GFP+Cow-miRNA-1*) in the D side of the wing disc, endogenous Cow was not detected with the anti-Cow antibody (cyan) in D GFP^+^ cells (green) when compared to the V control side. This result demonstrates the specificity of the Cow antibody and the efficiency of the RNA interference constructs. This knockdown could be observed at both the Ap and Ba surfaces. Less Cow protein was detected at the V side near the D-V border, suggesting that Cow may move toward the D cells from the V cells near the border. (F) Anti-Flag detected bands at both 100 kDa and 75 kDa of Flag-Cow in the cell pellet and supernatant of S2 cells transfected with *UAS-SP-Flag-Cow*. When the two putative glycosylation sites were mutated (in Flag-Cow-mSG1+2), the 100-kDa band was dramatically reduced and the 75-kDa band was enhanced. This suggested that the 100-kDa band represents the HS-modified form, whereas the 75-kDa band represents the unmodified form. Anti-tubulin was used to demonstrate the purity of the supernatant.
(EPS)

Figure S2 **Genetic interaction of** ***cow*** **and** ***wg*** **.** (A–D) Adult wing of (A) *tub-Gal4* (representing WT), (B) *MS1096>Cow-dsRNA*, (C) *tub>Cow*, and (D) *tub>Testican-2*. The phenotypes of *MS1096>Cow-miRNA-1* (unpublished data), *MS1096>Cow-miRNA-2* (unpublished data) and *MS1096>Cow-dsRNA* were similar. There were ectopic chemosensory bristles along or near the A and P margins (arrowheads in B′) with 60–85% penetrance. (C–D) Overexpression of Cow or human Testican-2 produced extra vein tissue (arrows) and loss of chemosensory bristles at the wing margin (arrowheads in C′ and D′ compared with WT in A′). The results are summarized in E. (E) Chemosensory bristle numbers from *tub-Gal4*, *tub>Cow* and *tub>Testican-2*. Overexpression of Cow or Testican-2 reduced the numbers of chemosensory bristles on the adult wing margin. The differences in comparison with the control, *tub>Gal4*, were statistically significant (**: p<0.001). (F) The adult phenotype of the *cow^5Δ^* mutant clone (*ap-Gal4/UAS-FLP; FRT82B ubi-GFP Minute/FRT82B cow^5Δ^*) that caused the wing-loss phenotype. (G) Adult phenotypes of *MS1096>DFz2N*, *MS1096>DFz2N+2XCow*, *MS1096>DFz2N+2XCow-dsRNA* and *MS1096>DFz2N+Cow-miRNA-*, which each led to wing loss. (H) Adult wing phenotype of *MS1096>DFz2*, *MS1096>DFz2+2XCow*, *MS1096>DFz2+2XCow-dsRNA* and *MS1096>DFz2+Cow-miRNA-1*.
(EPS)

Figure S3 **Cow affects the expression of Wg target genes.** (A–E) Sens expression (green) along the D-V border of the wing disc in (A) WT, (B) *MS1096>DFz2*, (C) *MS1096>2xCow*, and (D) *MS1096>2xCow-dsRNA*. The number of Sens^+^ cells in each group is summarized in (E). The results in B-D are significantly different from that of the WT (A).
(EPS)

Figure S4 **HS-modified Cow can stabilize Wg.** (A–C) S2 cells were transfected with the HA-Wg construct. Conditioned medium with secreted HA-Wg was then added back to new S2 cells transfected with control or *Cow-dsRNA* vectors. The HA-Wg and Cow in the culture supernatant were then examined by western blot. (A and C) HA-Wg was maintained at similar levels in the supernatant of empty control vector cells over 48 h (95% at 12 h, 94.5% at 24 h, 93% at 48 h relative to 100% at 0 h). When Cow was knocked down with the *Cow-dsRNA* vector, the HA-Wg level in the supernatant gradually decreased (88.5% at 12 h, 72% at 24 h, 53% at 48 h relative to 100% at 0 h). (A and B) Cow protein level decreased gradually in the supernatant after *Cow-dsRNA* transfection (75 kDa: 94% at 12 h, 81.5% at 24 h, 59.5% at 48 h relative to 100% at 0 h, 100 kDa: 96% at 12 h, 84% at 24 h, 59% at 48 h relative to 100% at 0 h), but Cow was maintained at the same level in cells transfected with the empty control vector (75 kDa: 94.5% at 12 h, 99% at 24 h, 101.5% at 48 h relative to 100% at 0 h, 100 kDa: 99.5% at 12 h, 99% at 24 h, 99% at 48 h relative to 100% at 0 h) (n = 3). Comparison of 75 kDa intensity at different time of dsRNA treatment with control (time 0): *, p<0.01, **, p<0.001, ***, p<0.0001. Comparison of 100 kDa intensity at different time of dsRNA treatment with control (time 0): ##, p<0.001, ###, p<0.0001. (D) S2 cells were transfected with empty control or Wnt5a-HA vectors. Wnt5a was detected in the cell pellet and culture supernatant with an anti-HA antibody 48 h after transfection. (E) S2 cells were transfected with empty control or SP-Flag-Testican-2 vectors. SP-Flag-Testican-2 was detected in the cell pellet and culture supernatant with an anti-Flag antibody 48 h after transfection. Anti-tubulin showed that the supernatant was not contaminated with cells. (F–G) Culture supernatants from S2 cells transfected with control vector, Wnt5a and SP-Flag-Testican-2, respectively, were mixed in different combinations. (F) When anti-HA was used for immunoprecipitation, anti-Flag detected SP-Flag-Testican-2. (G) When anti-Flag was used for immunoprecipitation, anti-HA detected Wnt5a-HA. For (C), n.s., not statistically significant, **, p<0.001, ***, p<0.0001.
(EPS)

Figure S5 **Cow enhances the rate of extracellular movement of Wg.** (A) In monitoring the speed of Wg movement, we used *en-Gal4*+*tub-Gal80^ts^* to drive *UAS-GFP* expression in an inducible fashion. The *en-Gal4*+*tub-Gal80^ts^>GFP* larvae were raised at 17°C and shifted to 30°C to inactivate Gal80^ts^, thereby inducing GFP expression. The GFP signal in the P compartment became weakly detectable starting at 3 h, was strong at 6 h, and reached full expression at 16 h after the temperature shift. Similar kinetics were observed for *UAS-GFP* driven by the *ap-Gal4* (unpublished data). (B–J) The rate of movement of endogenous Wg was estimated by blocking endocytosis (expressing Shi^ts^ and shifting temperature from 17°C to the non-permissive 32°C for 3 h for late third instar wing disc) and then relieving the endocytic block (by shifting back to 17°C) to initiate Wg distribution. Wg distribution was examined immediately or 60 min after the shift back to 17°C. *nub-Gal4* was used to drive expression in the wing pouch area of the wing disc. (B–E) The Wg distribution ranges in the Ap (B and D) and Ba (C and E) surfaces of *nub>Shi^ts^+lacZ* at 0 min (B–C) and 60 min (D–E). Significant numbers of Wg puncta were still observed at the Ap surface (B), but not at the Ba surface (C), away from its D-V source immediately after the return to the permissive 17°C. The distance of Wg puncta farthest from the Wg source was averaged to generate the Wg distribution range. (F–I) With knockdown of Cow, the Wg distribution at the Ap (F and H) and Ba (G and I) surfaces of *nub>Shi^ts^+Cow-miRNA-1* was scored at 0 min (F–G) and 60 min (H–I). (J) The apparent rates of movement for endogenous Wg at the Ap and Ba surfaces are summarized for *nub>Shi^ts^+lacZ* and *nub>Shi^ts^+Cow-miRNA-1*. (K–O) The Wg (white), Dll (red) and wing pouch size were measured at the indicated times 2 h after egg collection and cultured at 25°C (K–K‴, n = 11) for 70–72 h, (L–L‴, n = 7) 76–78 h, (M–M‴, n = 14) 82–84 h, (N–N‴, n = 11) 94–96 h and (O–O‴, n = 10) 110–112 h in *nub>GFP* (representing WT). (P) The distances between the wing pouch margins and the D-V boundary were measured to calculate the rate of growth. White bar, 20 µm. In WT, Wg was widely detected at low levels in the wing disc at 70–72 h AEL and gradually became high at the D/V boundary between 76–78 h and 82–84 h AEL (K-M; Alexandre et al., 2014). In addition, Wg spread from the D/V source toward the border of the wing pouch (Figure S5, N–O). Dll was undetectable until 84 h AEL, and its range expanded from 96 h to 112 h AEL (K″–O″). Therefore, we focused our analysis on the period from 84–112 h AEL.
(EPS)
